# Directing Salt‐Drop Movement on Mesoporous Silica Films

**DOI:** 10.1002/smsc.70340

**Published:** 2026-07-16

**Authors:** Laura Czerwenka, Chirag Hinduja, Lisa Balonier, Rüdiger Berger, Annette Andrieu‐Brunsen

**Affiliations:** ^1^ Ernst Berl Institute for Technical and Macromolecular Chemistry Macromolecular Chemistry – Smart Membranes Technical University Darmstadt Darmstadt Germany; ^2^ Max Planck Institute for Polymer Research Mainz Germany

**Keywords:** aqueous salt solution, asymmetric airflow, capillary imbibition, contact angle, drop movement, evaporation, Marangoni flow, mesoporous silica layer, nanofluidics, wetting

## Abstract

Programmable drop sliding on surfaces is of interest for microfluidics, self‐cleaning surfaces, water harvesting, or drop nanoreactors. While drop movement along gradients on surfaces or on slippery surfaces has been demonstrated, achieving programmable movement of drops on nanoporous surfaces without structural or chemical gradients requires different mechanisms of symmetry breaking. Using a hydrophilic mesoporous film, we investigate direction‐controlled drop sliding of aqueous salt solutions. We observe drop sliding using an aqueous NaCF_3_SO_3_ solution drop together with symmetry breaking through local airflow. The direction in which the drop moves is controlled by evaporation‐induced, asymmetric salt distribution. We investigate how the macroscopic static contact angle below 10°, the use of a continuous airflow, and varying salt concentrations allow the speed, distance, and direction of the drop to be programmed. The motive force increased with increasing NaCF_3_SO_3_ concentration in the drop and reaches 7 µN for 5 µL drops. The tuning of the drop direction and speed, driven by asymmetric airflow and controlled by ion concentration and airflow intensity‐induced local evaporation, provides a new perspective and mechanistic approach to programmable fluid drop transport, especially on mesoporous surfaces.

## Graphical abstract

A new mechanism of drop movement is presented. Specific aqueous salt solution droplets move on mesoporous silica films in the presence of a local airflow. While their direction is controlled by the airflow, their speed is controlled by the salt type, salt concentration, and airflow intensity. Uphill movement was observed for tilt angles below 6°.

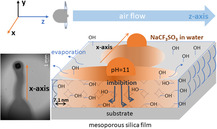

## 1 Introduction

The transport of fluids on surfaces plays a decisive role in many industrial and environmental processes [1, 2, 3]. The movement of drops on surfaces is particularly relevant in many technologies such as nano‐/microfluidics [4], water and energy harvesting [5], and electric fields [6]. Numerous studies have demonstrated the potential of drop movement through various mechanisms, including wetting gradients [7], electrostatic forces [8], and switchable surfaces [9]. Thereby, the strength of the gradient determines the drop motion. In order to enable programmable drop movement on a surface, symmetry breaking is required. One well‐established strategy is the use of wettability gradients, which can be achieved by fabricating chemically inhomogeneous surfaces [10]. Another one is the use of lubricant‐impregnated surfaces [11]. In general, strategies for optimizing drop movement using wettability can be divided into three strategies: application of driving force to a drop on nonsticking surfaces, formation of gradient surface chemistry/structure, or anisotropic surface chemistry/structure [12]. In addition to wettability gradients, a variety of alternative concepts and mechanisms have been developed to promote drop self‐transport [13, 14, 15]. These include designing ultraslippery surfaces [16] and slippery liquid‐infused porous surfaces (SLIPS) [17] as well as manipulating magnetic drops under external fields [18], and the use of body forces [19]. Within these categories, further mechanisms have been explored such as electrowetting [20, 21], Leidenfrost effects [22], and Marangoni‐driven flows, which also rely on locally varying interfacial forces [23, 24]. Further methods include electrohydrodynamic actuation [25] and various surface engineering strategies [26]. Drop movement on a solid surface can be driven by Marangoni flows and thus by the generation of surface gradients [27, 28, 29]. Marangoni flows can be categorized into thermal [30] and solutale [31, 32] Marangoni flows. This driving force can be generated, for example, by photothermal heating, surfactants, or condensation [33, 34, 35]. Surfactant‐ and/or salt‐induced effects lead to surface tension gradients, which in turn lead to Marangoni flows that influence the drop dynamics on and near an aqueous surface [36] or on solid surface [37]. Furthermore, capillary fluid flows and Marangoni‐driven flows can arise due to inhomogeneous molecular distribution patterns and, for example, control particle transport within a drop [27]. Recently, the self‐propulsion of a vaporizing drop on a polymer‐coated surface was reported. The self‐propulsion was ascribed to an asymmetric Marangoni vortices through a so‐called “push–pull” mechanism [28]. Recent work shows, how drop movement can be specifically controlled on so‐called slippery surfaces (SLIPS) using external forces [38]. The used external forces range from magnetic fields [39], self‐propulsion via wettability gradients in immiscible lubricant films [40], or capillary pinning of drops via lubricant meniscus interactions at microstructured SLIPS [41]. Due to their low contact angle (CA) hysteresis, SLIPS are relevant due to their anti‐icing and antibiofouling capabilities [11, 42, 43].

Thus, the physical mechanisms underlying drop movement are of paramount importance. Furthermore, the nature of the surface is a contributing factor to the dynamics of the drops [12, 44]. Superhydrophobic [45], hydrophobic [46], hydrophilic [47], and superhydrophilic [48, 49] surfaces represent the four main types of surfaces used for drop movement.

For example, Daniel et al. showed that many small drops are deposited together by saturated vapor on a cold water‐repellent surface and move analogously to Brownian molecular motion. However, when a surface tension gradient was added, the drops moved specifically to the lower CA side and moved faster than typical Marangoni flows [50]. Chakraborty et al. demonstrated a water drop moving ten times faster on a silicon surface with a chemically induced hydrophilicity gradient when the temperature increases by 40°C. In particular, the contact line represented the greatest frictional resistance, which decreased with increasing temperature [51]. Recently Xu et al. showed wetting behavior and water drop movement control on microstructured, hydrophilic surfaces which was regulated using vapor from another liquid with a lower surface tension. By switching the vapor environment, Marangoni forces induced by condensation enable reversible drop formation and recovery from previously imbibed liquid films [52]. Latthe et al. referred to hydrophobic porous silica surfaces based on methyltriethoxysilane (MTES), which were produced using a sol–gel process [53]. Upon adding trimethylethoxysilane (TMES), a minimum sliding angle of 9° is sufficient to allow the drop to slide with low adhesion.

In contrast to studies on drop movement on hydrophobic or hydrophilic structured surfaces, studies on drop movement on mesoporous surfaces remain scarce. The imbibition of fluids from a drop into a mesoporous layer was intensively investigated within recent years [54, 55, 56, 57, 58, 59]. Furthermore, Giménez et al. showed that water and oil drops move more easily on mesoporous surfaces than on nonporous materials [60]. This is ascribed to reduced pinning at the contact line, resulting in a lower sliding angle and higher sliding velocity. This mechanism differs from those observed in other surfaces such as superhydrophobic or oil‐based surfaces.

Sbeih et al. recently investigated how sliding of aqueous salt solution drops with different ion type and concentrations generate an electrical charge on a hydrophobic solid surface and how this differs from pure water [1, 61]. Low concentrations of salt led to a slight increase in surface charge, while high concentrations of salt showed the opposite effect. This decrease in charge was attributed to charge shielding due to increasing salt concentration and pH‐controlled charge regulation. A further study investigates the adhesion of aqueous salt solution drops to superhydrophobic surfaces at varying temperatures by subjecting the drops to an external airflow (critical velocity) [62]. Drops containing salt exhibited a stronger adhesion to the surface in comparison to water, thereby impeding the process of shedding. As temperatures decrease, this effect is intensified and detachment becomes more challenging. Pizarro et al. recently introduced drops on mesoporous surfaces as compartments enabling chemical communication between them via molecular exchange when their imbibition rings are in contact demonstrating the potential of drop nanoreactors [63].

Here, we show a programmable drop movement on hydrophilic mesoporous surfaces using aqueous NaCF_3_SO_3_ salt solution. The direction and speed of the drop movement is controlled by a local airflow. The cause of the drop movement is investigated. The role of evaporation, Marangoni flows through asymmetric evaporation and the resulting asymmetric salt distribution are discussed. The influence of varying the salt type, the salt concentration of an aqueous NaCF_3_SO_3_ solution and of the varying substrate tilt angle on the speed of drop movement is investigated. The friction force of a drop on a surface was analyzed using drop friction force instrument (DoFFI) measurements in dependence of the drop movement speed. Our results are of general relevance for sensing and microfluidics, drop compartment communication, or for water treatment, efficiency enhancement of water harvesting, or surface patterning [64, 65].

## 2 Experimental Section

### 2.1 Materials

Pluronic F127 and potassium perchlorite (KClO_4_) were purchased from Sigma‐Aldrich. Tetraethoxysilane (TEOS) and sodium trifluoromethanesulfonate (NaCF_3_SO_3_) were purchased from Alfa Aesar. Ethanol (absolute) was purchased from Merck Millipore. Potassium chloride (KCl) was purchased from superlco. Potassium phosphate (K_3_PO_4_) was purchased from acros organics. Fluorescein sodium was purchased from Carl Roth KG. All chemicals were used as received unless otherwise noted.

### 2.2 Preparation of Mesoporous Silica Films

Mesoporous silica films were prepared by dip coating, sol–gel chemistry, and evaporation‐induced self‐assembly (EISA) using a method adapted from Dunphy et al. [66]. Briefly, tetraethyl orthosilicate (TEOS) was used as the silica precursor, while Pluronic F127 acted as the structure‐directing agent. A precursor sol with the following molar ratios was used: 2.1 TEOS (9.76 mL): 0.02 Pluronic F127 (5.2 g): 34 H_2_O (12.7 mL): 40 ethanol (48 mL): 0.03 HCl (37%, 0.05 mL). The freshly prepared solution was directly used for dip coating onto silicon wafer substrates at ≈60% relative humidity (RH) and ≈23°C, using a withdrawal speed of 2 mm s^−1^. After deposition, the freshly formed films were kept at 60% RH for 1 h. A subsequent thermal posttreatment was applied, heating the films to 60°C within 10 min and keeping this temperature constant for 1 h, before heating to 130°C within 10 min and keeping this temperature constant for another hour. Subsequently, the temperature was increased to 350°C at a rate of 1°C min^−1^, followed by maintaining this temperature constant for 2 h. Once cooled to room temperature, the mesoporous silica films were rinsed with ethanol and stored under ambient conditions. Ellipsometry and data evaluation using the effective medium theory confirmed a mesoporous silica layer thickness of ≈600 nm and a porosity of ≈61 vol%.

### 2.3 Plasma Treatment (Activation)

To prepare mesoporous silica film with a low CA, the mesoporous film was activated using plasma generated with air being flown through water into the plasma chamber. Samples were in contact with the plasma for 2 min using a low‐pressure plasma equipment from Diener electronic; type: Femto; chamber volume approx. 1.7 liters; housing: W: 345, H: 220, D: 420 mm; plasma generator: 40 kHz/100 W, infinitely variable; generator frequency 100 kHz, 0–30 W; control: semiautonomous; process time via timer.

### 2.4 Preparation of Aqueous Salt Solutions

For the analysis of the drop movement and for the CA measurements, aqueous NaCF_3_SO_3_ solutions of varying concentrations (0.05, 0.1, 0.5, 0.75, 1.25 M) were utilized. In addition, aqueous solutions of KCl, KClO_4_, and K_3_PO_4_ in varying concentration of 0.05, 0.1, 0.5 M and Mg(CF_3_SO_3_)_2_ and NaCF_3_COO at a concentration of 0.5 M were used. With increasing NaCF_3_SO_3_ concentration, the solution pH value increased from 8 to pH 11. All solutions were prepared using 25 mL of ultrapure water (Milli‐Q, 18 MΩ·cm) and sonicated for 10 min to promote complete dissolution. The pH of each solution was subsequently measured. In the case of K_3_PO_4_, the pH was adjusted to 7.0 using concentrated H_3_PO_4_. The specific compositions of the salt solutions are shown in Table S1.

### 2.5 Grafting of SBDC on Mesoporous Films

For SBDC functionalization, a solution of 46.5 mg SBDC [67] dissolved in 54 ml of dry toluene (2.3 mM) was prepared and purged with nitrogen for 10 min. The previously plasma‐activated mesoporous films were placed in another flask and heated under vacuum to 350°C to remove excess water. The SBDC solution was then poured into the flasks filled with plasma‐activated mesoporous films and treated at 80°C for 1 h. Finally, the SBDC‐functionalized films were rinsed with toluene and ethanol, extracted for 10 min each, and air‐dried.

### 2.6 PMETAC (poly[2‐(methacryloyloxy)ethyl] trimethylammonium chloride) Functionalization of Mesopoporous Silica Films via PET RAFT Polymerization

For PMETAC functionalization, a visible‐light‐induced oxygen tolerant PET RAFT polymerization was used. The monomer METAC, photocatalyst ZnTPP, and solvent DMSO with following molar ratio were used, as described in our previous work [68]: [monomer]: [ZnTPP] = [500]: [0.025] in 10 mL DMSO (10 mL per 1 mg ZnTPP) using the monomer concentration of 2.7 mol L^−1^. The monomer solution was treated in an ultrasonic bath for 5 min before being transferred to the Petri dish with SBDC‐functionalized films. The films were then irradiated for 5 minutes at 405 nm (Hoenle, 5%, 23.25 mW/cm^2^), washed with water (Milli Q, 18 ohms) and ethanol, extracted in water for 60 min, and air‐dried.

### 2.7 Contact angle Measurements

Macroscopic static CA measurements were performed using an OCA 35 goniometer (DataPhysics Instruments, Filderstadt, Germany) equipped with SCA 4.5.2 software, employing the sessile drop method under ambient laboratory conditions (T = 23°C, RH = 50%). A droplet volume of 2 μL was applied, and the CA was determined by fitting the drop profile using the software's integrated approximation algorithm. Mean static CA values were calculated from measurements of at least two or three individual drops. For CA analysis, PMETAC‐functionalized and mesoporous silica films were incubated in the respective 0.1 M salt solutions for 30 min, rinsed with ethanol, dried with compressed air, and subsequently measured using the same aqueous salt solution. In contrast, CA measurements with water and 0.5 M NaCF_3_SO_3_ were conducted without prior incubation of the films.

### 2.8 Drop Movement Measurement

To analyze the drop movement on top of a mesoporous silica film, a Topview Video System TVS‐C (Dataphysics OCA35, software SCA 4.5.2 Build 1052) with collimated coaxial illumination was used to capture the imbibition ring around the moving drop by detecting changes in the refractive index and the drop movement by itself. A drop volume of 1 μL was placed onto the mesoporous films (used solutions 0.05, 0.1, and 0.5 M) (Table S1) and the videos were recorded at ≈11 frames per second (fps). To control the drop movement through an airflow, an EasyAcc hand‐fan with four different speed options was used. For drop movement experiments, two speed options with 2000 and 4500 rpm were used corresponding to 0.21 and 0.3 m/s at the drop position with a distance of 4 cm. The airflow was measured using a TA 405i thermal anemometer with a measuring range from 0 to 30 m/s at fixed distances in a climate room with a relative humidity of 52.1% and a temperature of 23°C. The exact airflow characteristics at different positions and distances are shown in Figures S8 and S9, Tables S2 and S3. The hand‐fan was placed ≈4 cm next to the film with the drop, so that both are not in direct contact (Figure S7). For data evaluation (speed, distance of drop movement, and pictures of salt distribution), IMAGEJ was used. As fluorescent dye, a spatula tip of fluorescein was placed in 10 mL of 0.5 M NaCF_3_SO_3_ solution without exact concentration adjustment. Two µL of the fluorescein mixture was placed on the mesoporous (plasma‐activated) silica film to visualize the path the drop was moving.

### 2.9 Drop Movement Measurement with Tilt Angle

The mesoporous film was placed on an OCA 35 goniometer (DataPhysics Instruments, Filderstadt, Germany) equipped with SCA 4.5.2 software. The device is able to rotate to different tilt angles. For the measurement, tilt angles with 1°–6°, 12°, and 20° were used. A 1 µL aqueous 0.5 M NaCF_3_SO_3_ drop was placed onto a mesoporous film, and then, the device was tilted by 1°–6°, 12°, or 20°. After the angle was set, the fan was set on 2000 rpm or 4500 rpm and placed on the opposite tilt direction. A Topview Video System TVS‐C (Dataphysics OCA35, software SCA 4.5.2 Build 1052) with collimated coaxial illumination was used to analyze the drop movement.

### 2.10 Drop Friction Force Measurements

We performed drop friction force measurements on our mesoporous films using DoFFI [69]. DoFFI setup consists of an XY stage, a thin glass capillary which acts as a force sensor and a complementary metal–oxide–semiconductor (CMOS) camera. The dimensions of the glass capillary were 0.05 × 0.5 × 50 mm^3^. One end of the glass capillary was fixed in the capillary holder. On the other end of the capillary, a metallic ring of diameter 1 mm was attached with the help of an epoxy (UHU Plus instant). The spring constant of the force sensor used in our measurements was 90 ± 5 µN/mm. The sample was placed on the XY stage and was fixed on the stage with the help of clips. Then, we placed a 5 µL liquid drop on mesoporous films and attached the force sensor to the drop. For the DoFFI measurements, drops smaller than 5 µL are hardly feasible, which is why the minimum drop volume was used here to avoid deviating even further from the drop volume of the other measurements. Then, we moved the stage in forward and backward motion at a speed. We used two different stage speeds of 1 and 10 mm/s in our experiments. Using the CMOS camera, we recorded deflection of the sensor from its reference position. The images were acquired at a speed of 30 frames per second with resolution of each image being 6.7 µm/px. To get the friction force, we multiplied the spring constant of the sensor with the deflection of the sensor. We averaged the friction force in the kinetic regime.

### 2.11 Drop Motive Force Measurement

The motive force is the external force acting on the drop in the presence of a fan and leads to sliding of drops. We used the drop friction force instrument to measure motive forces acting on a drop. The drop adheres to the force sensors and any additional motive force acting on the drop deflects it. Thus, the deflection of the force sensor balances the motive force up to a force which leads to the detachment of the drop from the force sensor. To measure this motive force, a 5 µL drop of NaCF_3_SO_3_ salt was deposited on the plasma‐activated mesoporous sample. This drop was immobilized by the force sensor equipped with a 1‐mm metallic ring. To fit the fan into the device, we placed the center axis of the fan (EasyAcc hand‐fan) at a distance of ≈20 mm from the drop. The fan speed was 4500 rpm. Due to an airflow, the drop's motive force is in the direction of the fan and perpendicular to the fan axis. As a result, the capillary deflected from its reference, i.e., undeflected, position. We recorded the deflection of the capillary in side view with a CMOS camera. The images were acquired at a speed of 10 frames per second for 180 sec. The image resolution for each image was 6.7 µm/px. Then, we calculated the deflection of the capillary in mm from its reference position, using MATLAB 2021b. The deflection was multiplied with spring constant of the capillary force sensor to obtain the motive force.

## 3 Results and Discussion

**FIGURE 1:**
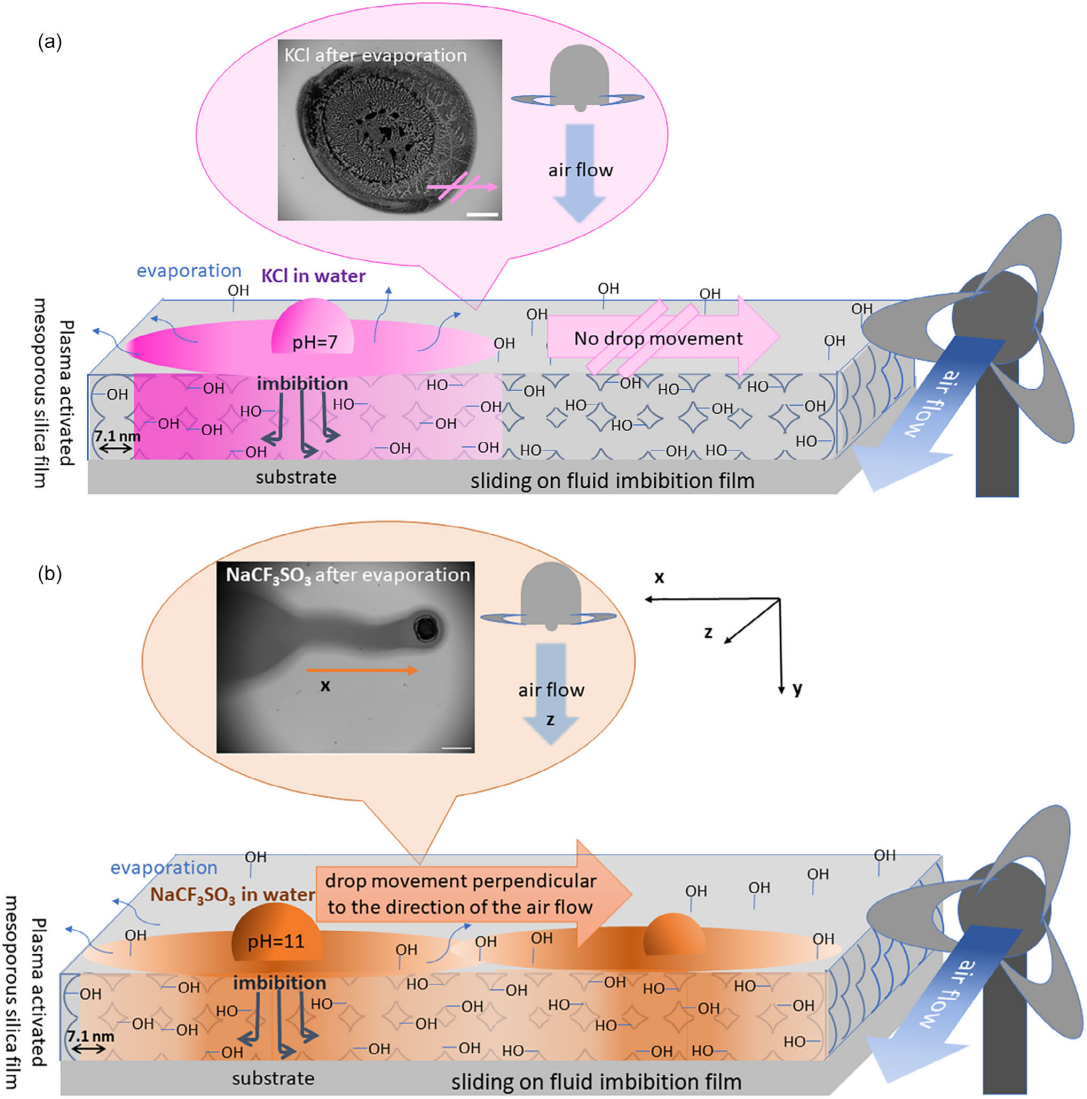
Schematic visualization of (a) no‐drop movement of aqueous (0.5 M) KCl (pink) and (b) drop movement perpendicular to the direction of and towards the airflow of aqueous (0.1 M) NaCF_3_SO_3_ drop (orange) on a plasma‐activated mesoporous silica surface. The *z*‐axis represents the airflow direction, while the droplet's moving direction advances along the *x*‐axis. The scale bar in (a) and (b) equals 1 mm.

**FIGURE 2:**
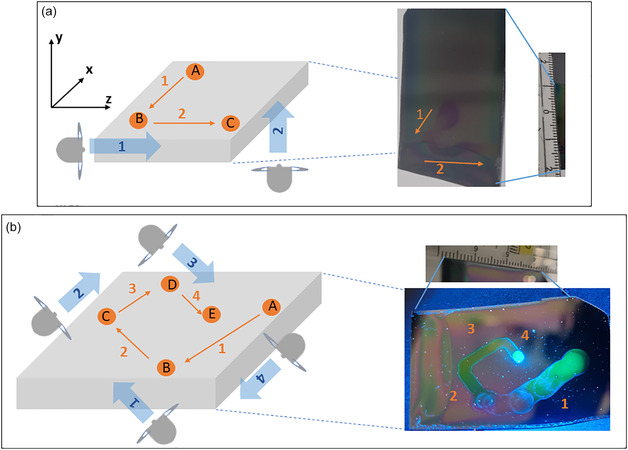
(a) Schematic representation of the drop movement directional control using an airflow strength of 2000 rpm for 1 µL 0.5 M NaCF_3_SO_3_ drop on a plasma‐activated mesoporous silica film. The drop at position A moves to position B (along the *x*‐axis) using the airflow 1 (blue arrow, *z*‐axis). Thereby, the drop always moves toward the airflow direction and thus to position B (C) using the airflow direction 1 (2), respectively. (b) Schematic representation of how a 2 µL aqueous 0.5 M NaCF_3_SO_3_ drop containing fluorescein on a plasma‐activated mesoporous silica film is guided by the airflow strength 1 (2000 rpm). The drop on the starting position A moved to position B through airflow direction 1. Subsequently, the drop on position B moved to position C through airflow direction 2. Finally, the drop on position C moved to position D by using airflow direction 3, turning toward position E through airflow direction 4. The fluorescein used in this image serves solely to visualize the direction in which the drops flow. It appears slightly green when illuminated by visible light. The drop direction programming was not affected by the presence of fluoresceine as can be seen by comparing Figure 2a,b. Measurements were performed under constant humidity (≈60%) and temperature (≈23°C).

Mesoporous silica films were prepared on silicon wafer substrates using sol‐gel chemistry and EISA [70]. Pluronic F127 was employed as the mesopore template, and tetraethyl orthosilicate (TEOS) served as the silica precursor. The resulting mesoporous films exhibited a thickness of ≈600 nm, an average pore size of ≈7.1 nm, and a porosity of 61 vol%, as previously reported [65]. Upon deposition of a drop on top of the mesoporous film, the fluid imbibes into the mesoporous film by capillary imbibition. This imbibition results in a circular fluid filled zone around the deposited drop which is called the imbibition ring [65] as shown in Figures 1 and 2. Thereby, evaporation occurs from the drop as well as from the imbibition ring [65, 71].

**FIGURE 3:**
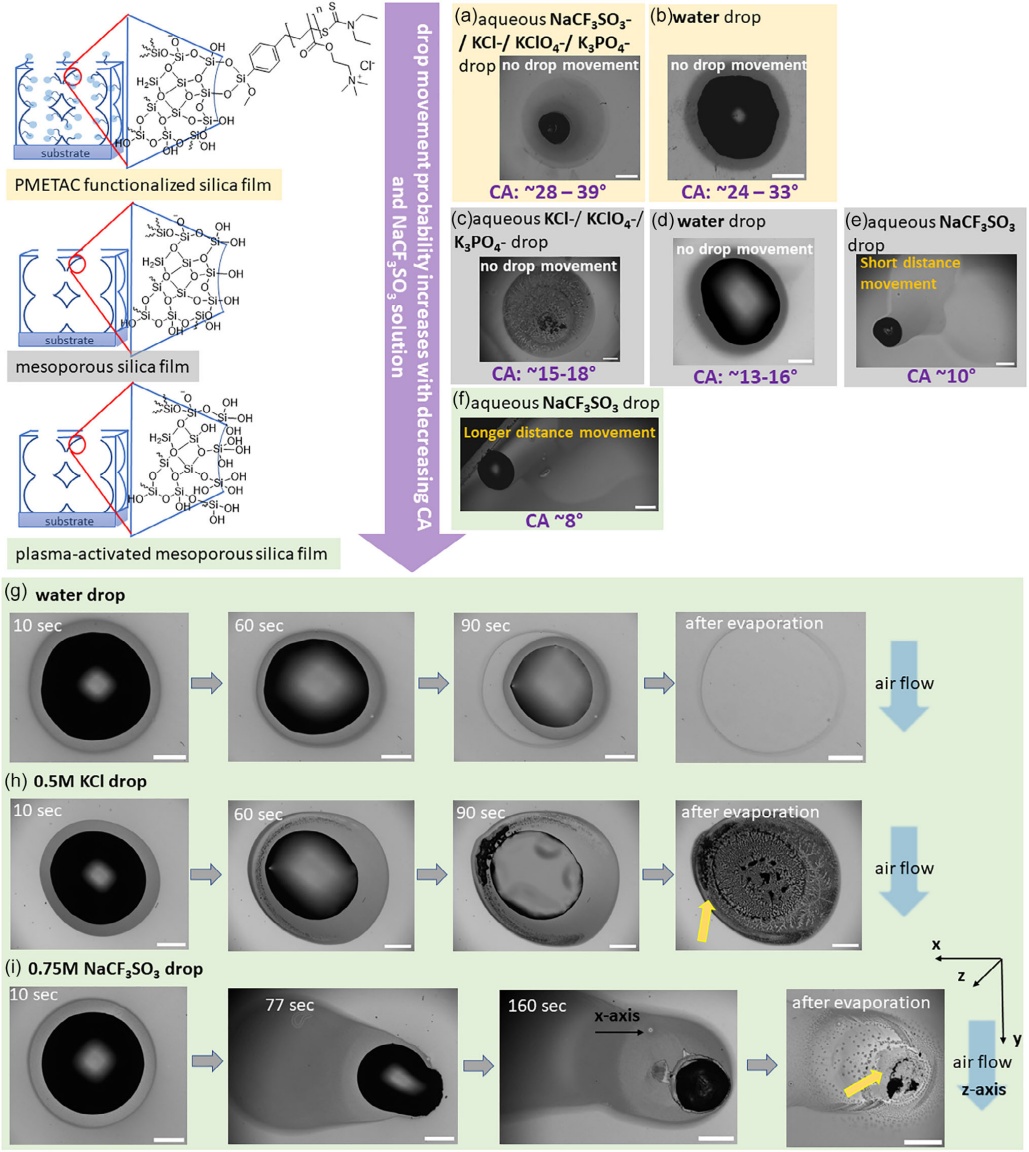
No‐drop movement was observed for PMETAC‐functionalized silica films (yellow) with a CA between 24° and 39° using (a) aqueous salt solution drops of 0.1 M aqueous KCl, KClO_4_, K_3_PO_4_, NaCF_3_SO_3_ and (b) water drops. As well no drop movement was observed for mesoporous silica films (gray) with a CA between 13° and 18° using (c) aqueous (0.1 M of KCl‐, KClO_4_−, and K_3_PO_4_−) drops or (d) water drops. For drop movement experiments on mesoporous silica films with (e) aqueous 0.1 M NaCF_3_SO_3_ solution drops a slight drop movement for films with a CA of ≈10° and a good movement on (a,f) plasma‐activated mesoporous silica film with a CA of ≈8° was observed. The CA measurements for NaCF_3_SO_3_, KCl‐, KClO_4_−, and K_3_PO_4_ salt solutions were performed on incubated PMETAC functionalized films (a) and incubated mesoporous films (c,e) using the corresponding salt solution for incubation prior to CA measurements. (g) Using a pure water drop, no‐drop movement was observed even when using an airflow intensity of 4500 rpm analyzing a 1 µL drop of water after 10, 60, and 90 s and after complete drop evaporation on a plasma‐activated mesoporous silica film. (h) As well an aqueous 0.5 M KCl drop analyzed after 10, 60, and 90 s and after complete evaporation on a plasma‐activated mesoporous silica film did not show any drop movement. (i) Drop movement experiments with airflow intensity of 2000 rpm analyzing 1 µL drops of 0.75 M NaCF_3_SO_3_ analyzed after 10, 77, and 160 s and after complete drop evaporation on plasma‐activated mesoporous silica film clearly show drop movement. Measurements were performed under constant humidity (≈60%) and temperature (≈23°C), Scale bar = 1 mm. The used salt solution components are shown in Table S1.

When depositing a 2 µL drop of water or aqueous salt solutions (KCl, KClO_4_, K_3_PO_4_) on mesoporous films, a macroscopic static CA of 13°–18° was observed (Figure 3c,d). For poly[2‐(methacryloyloxy) ethyltrimethyl ammonium chloride] (PMETAC)‐functionalized mesoporous silica films measured with water or aqueous salt solution drops (NaCF_3_SO_3_, KCl, KClO_4_, K_3_PO_4_), a CA in a range from 24° to 39° was observed (Figure 3a,b).

Interestingly, some drops start moving along the surface of the mesoporous film when being in contact with a local, asymmetric airflow (Figure 1b) while others do not (Figure 1a). This movement of a drop was monitored using a top‐view camera.

The drop position and motion can be systematically controlled by varying the position and strength of the airflow with respect to the drop position when using the fluorinated salt NaCF_3_SO_3_. In addition, the initial NaCF_3_SO_3_ concentration within the drop plays a role (Figure 2). The drop moves always orthogonal toward the airflow. If the airflow is for example oriented in *z‐*direction the drop always moves along the *x*‐axis orthogonal toward the *z*‐axis and thus toward the airflow as shown in Figures 1b and S7. In Figure 2a, the drop on position A is moving to drop position B using the airflow direction 1. By changing the airflow direction 1 to direction 2, the drop moved from position B to drop position C (Figure 2a). The corresponding experimental traces were visualized by refractive index changes. In addition, the corresponding experimental traces were visualized by adding the dye fluorescein into the drop (Figure 2b). The drop is directed by the orientation of the airflow, allowing, for example, the creation of patterns on a mesoporous film (Figure 2b). The overall path length the drop was moving, was dependent on the drop volume and on the evaporation. With increasing drop volume of up to 2 µL, a longer distance of drop movement of about 3.3 cm (Figure 2b) as compared to 2.3 cm for a 1 µL (Figure 2a) drop was obtained under the applied conditions.

In the following, the dependence of moving direction and related motive force on airflow intensity, salt type, surface type, and salt concentration is systematically analyzed.

### 3.1 Mesoporous Silica Functionalization and Salt Type

When comparing PMETAC‐functionalized mesoporous silica films (Figure 3a,b), unmodified mesoporous silica films (Figure 3c–e), and plasma‐activated mesoporous silica films (Figure 3f), drop movement was only observed for unmodified mesoporous silica films and for plasma‐activated mesoporous films. Thus, drop movement was only observed for mesoporous silica films with a macroscopic static CA below ≈10°. Furthermore, drop movement was only observed for aqueous NaCF_3_SO_3_ salt solution drops (Figure 3e,f) but not for a drop of aqueous solution of KCl, KClO_4_, or K_3_PO_4_. Drop movement was as well not observed for dense silica films without mesopores (Figure S1). This supports the observation that airflow‐induced drop movement using NaCF_3_SO_3_ is only possible when using mesoporous films. The observed speed of drop movement thereby suggests that the drop moves on liquid filled mesopores as the moving speed is slower than the speed of capillary fluid imbibition into the mesopores. Thereby, the movement starts at a specific time after drop deposition and the direction of movement as well as the traveled distance before drop evaporation varies.

In case of a plasma‐activated mesoporous film in contact with an asymmetric airflow at one side of the drop, the imbibition ring became asymmetric and was broader at the side close to the airflow. This was as well observed for KCl drops on plasma‐activated mesoporous films (Figure 3h), as well as for NaCF_3_SO_3_ solution drops on a mesoporous film which was before incubated in aqueous NaCF_3_SO_3_ solution (Figure S2). This imbibition zone broadening indicates that the airflow locally enhanced evaporation. A constantly increasing imbibition ring length upon locally enhanced evaporation indicates locally increasing ion concentration within the imbibition ring and thus further osmotic fluid pumping as we discussed in our previous work [65]. Furthermore, this asymmetrically enhanced evaporation probably induces Marangoni flows within the drop. This hypothesis is supported by analyzing the precipitated salt distribution after complete evaporation of the drop. Analyzing the salt distribution after complete fluid evaporation (Figure 3), an asymmetric salt distribution (Figure 3h,i yellow arrows) was observed for KCl as well as for NaCF_3_SO_3_ (Figures S2 and S3). Salt precipitation within the imbibition ring was already observed shortly after the fan started at the drop side opposite to the airflow for 0.5 M KCl solution drops. The same observation was made for 0.5 M NaCF_3_SO_3_ drops on a salt solution incubated surface with a CA higher than 10° (Figure S2). After complete evaporation, the highest precipitated salt amount was observed at the side of the drop having been located opposite to the airflow close to the three‐phase contact line (0.5 M KCl Figure 3h and 0.75 M NaCF_3_SO_3_ Figure S3). This indicates locally increased salt concentrations at the side opposite to the airflow probably due to evaporation‐induced flows within the drop. That the imbibition ring remains smaller at this side of the drop may be explained by the salt being precipitated which then reduces the dissolved concentration and with this the osmotic pressure within the imbibition ring at this side of the drop [65].

When using fluorinated salts (e.g., NaCF_3_SO_3_), an asymmetric salt distribution may result into an asymmetric wettability of the drop. This again may be the driving force to induce drop movement to the direction of locally lower salt content which is perpendicular to the direction of the airflow. This is supported by the observation, that 0.5 M NaCF_3_SO_3_ drops do not move on mesoporous films, which have been incubated into an aqueous 0.5 M NaCF_3_SO_3_ solution and which then show a CA higher than about 10° (Figure S2). Thus, a certain CA difference seems to be necessary.

### 3.2 Moving Direction and Tilted Substrate

**FIGURE 4:**
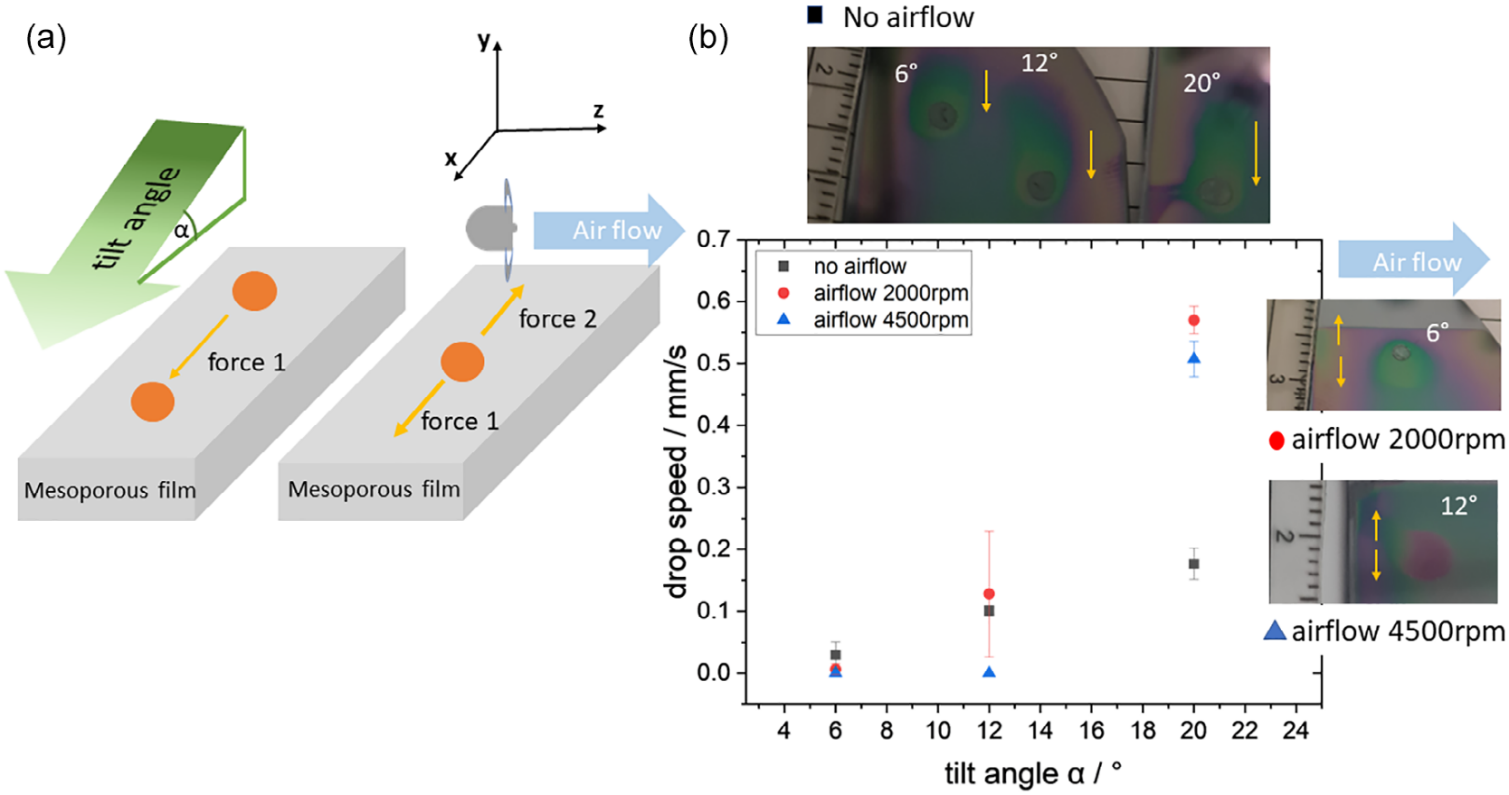
(a) Schematic representation of drop movement at different tilt angles α. Drop sliding down the substrate following gravity  can be completely suppressed through a certain airflow intensity. The airflow direction is defined as the *z*‐axis, while the droplet moves along the *x*‐axis. Depending on the airflow intensity, this motion is directed either toward the *z*‐direction or away from it. (b) Different drop speeds while using a 0.5 M aqueous NaCF_3_SO_3_ solution, no airflow (black), airflow strength 1 (2000 rpm, red), and airflow strength 2 (4500 rpm, blue) on mesoporous films. Images represent drop behavior with different tilt angles of 6°, 12°, and 20° while using no airflow, 2000 and 4500 rpm. The standard deviation was represented by the error bars and was determined from two or three individual drops measured on one or two substrates. Measurements were performed under constant humidity (≈60%) and temperature (≈23°C).

To further support the programmable movement of NaCF_3_SO_3_ drops on mesoporous films driven by symmetry breaking originating from the asymmetric airflow, we investigated drop movement in dependence of substrate tilt angle α (Figure 4a). Tilt angles of 6°, 12°, and 20° were used. The tilt angle α was adjusted before the drop was applied on the film. In the absence of airflow, the drop was sliding down the film and the speed increased with increasing tilt angle, as expected for gravity‐driven drop sliding (Figure 4b, black). When adding airflow through a fan at the upper substrate side with the flow direction being perpendicular to the substrate length, drop movement was significantly affected. The drop movement down the substrate following gravity was even completely suppressed through a certain airflow intensity or fan speed. For example, at a fan speed of 2000 rpm combined with a tilt angle of 6° (Figure 4b red) as well as for a fan speed of 4500 rpm (5.6 m/s, Table S3) and a tilt angle of 6° or 12°, no‐drop movement was observed (Figure 4b blue). For a higher tilt angle of 12° and an asymmetric airflow at 2000 rpm as well as for a combination of 20° tilt angle and 4500 rpm, the drop moved again down the substrate, indicating that gravity again determines the drop movement. This shows the balance between symmetry breaking by local airflow inducing drop movement and gravity directing drop movement to the opposite direction in this experiment. Additionally, with a tilt angle of 6° (2000, 4500 rpm) and 12° (4500 rpm), the drop was able to move parallel to airflow away from fan position (Figure S4). For tilt angles below 6°, we observed drop movement uphill toward the airflow (Figures S14 and S15). From tilt angles of 6° and higher, no‐drop uphill movement was observed anymore. This transition tilt angle of around 6° corresponds to the transition tilt angle estimated by considering the motive force and the combined resistance of body force and friction force. Assuming that the drop can move uphill when the motive force is higher than the combined resistance of body force and friction force, we estimated a transition tilt angle of 5.6° for plasma treated substrates with an advancing CA below 10° (Figure 3f). This rough estimation fits well to the observed data (Figure S14). For drop movement parallel to the airflow, the speed was defined as 0 (Figure 4b). At a tilt angle of 20°, we observed higher drop speed down the substrate in the presence of airflow as compared to the drop speed without airflow (Figure 4b, red, blue). Analyzing the speed increase with increasing tilt angle, a linear increase of the drop speed with increasing tilt angle is observed in the absence of airflow. In the presence of the asymmetric airflow, the drop speed does not linearly depend on the tilt angle, indicating a multiparameter influence which we ascribe to asymmetric evaporation‐ and salt distribution as well as induced flows within the drop in addition to gravity. An almost similar experimental setup with different tilt angles was demonstrated by Sartori et al. using Newtonian drops subjected to vertical oscillations [72].

### 3.3 Moving Distance, Speed, and Needed Force

**FIGURE 5:**
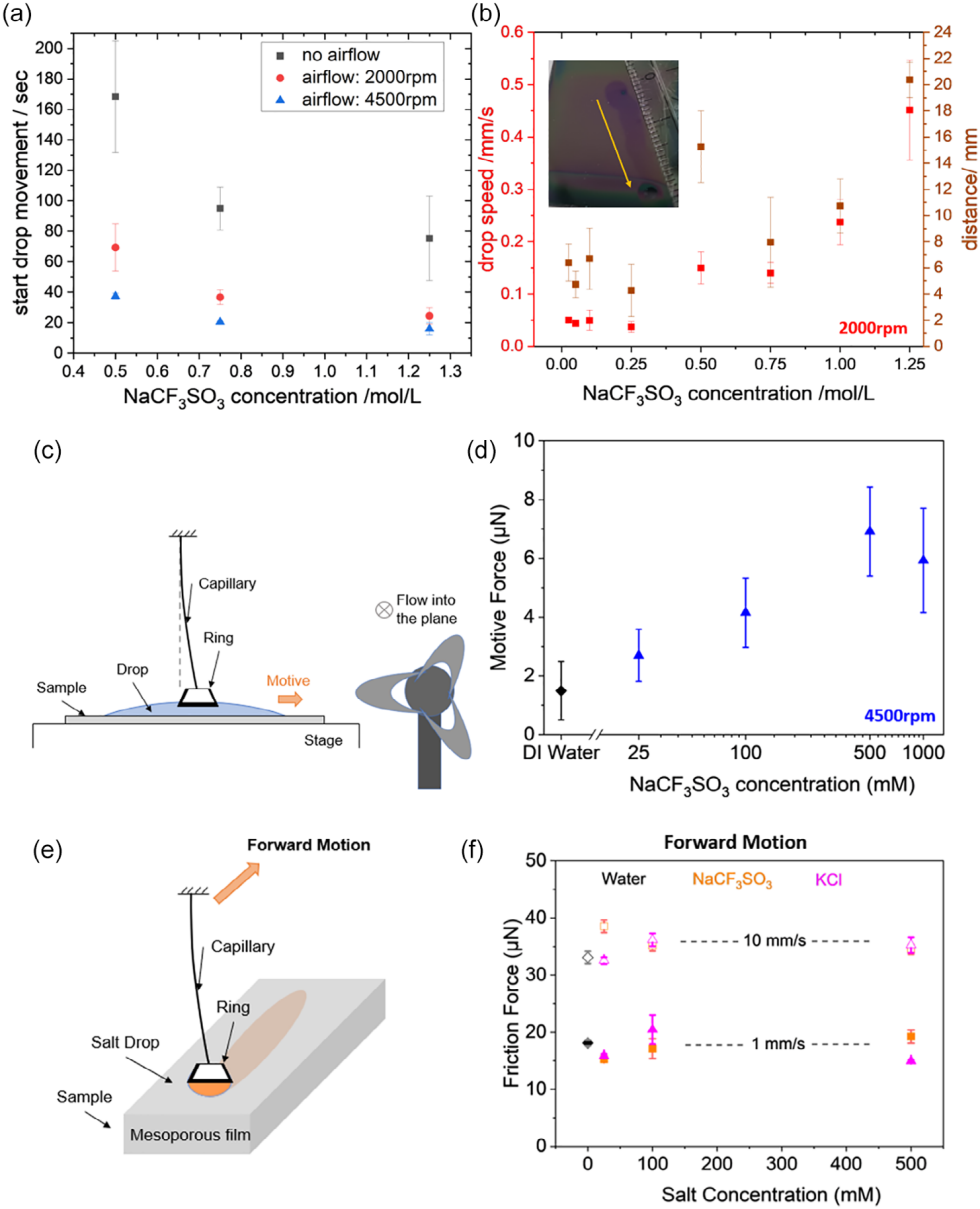
(a) Start time of drop movement on plasma‐activated mesoporous silica films with increasing 1 µL NaCF_3_SO_3_ drop concentration (0.5, 0.75, and 1.25 M) and increasing airflow intensity of 0 (black), 2000 (red), and 4500 rpm (blue). (b) Drop speed and distance of drop movement with increasing (0.025, 0.05, 0.1 0.25, 0.5, 0.75, and 1.25 M) 1 µL NaCF_3_SO_3_‐drop concentration on plasma‐activated mesoporous silica films at an airflow of 2000 rpm. The picture was recorded for 1 µL 1.25 M NaCF_3_SO_3_ drop volume. (c) A schematic illustration of drop motive force measurements on plasma‐activated mesoporous films using (d) water (black), 0.025, 0.1, 0.5, and 1 M aqueous NaCF_3_SO_3_ salt solution (blue) and an airflow of 4500 rpm. (e) Schematic representation of a drop friction force measurement, without airflow. A 5 µL water or aqueous salt (NaCF_3_SO_3_ or KCl) drop is placed on the capillary and is moved forward on top of the mesoporous film. Average kinetic friction force during (f) forward of a 0.025, 0.1, and 0.5 M KCl (pink)‐, NaCF_3_SO_3_ (orange), and water (black) drop on mesoporous silica film. The error bars were determined from three independent drops measured on one or two substrates. Measurements were performed under constant humidity (≈60%) and temperature (≈23°C).

To systematically investigate the time until the drop starts moving as well as to investigate the moving direction, speed, and distance, a drop of 0.1 M aqueous NaCF_3_SO_3_ solution was placed on top of the plasma‐activated mesoporous silica film (Figures 1 and 5). Although the pH varies with increasing NaCF_3_SO_3_ concentration, pH does not seem to be detrimental for drop movement as other salt solutions with similar pH do not show drop movement (Figure 3).

The time between drop deposition and initial drop movement was analyzed for different NaCF_3_SO_3_‐drop concentrations (0.5, 0.75, and 1.25 M, Figure 5a) and varying airflow intensities (0, 2000, and 4500 rpm). With increasing NaCF_3_SO_3_ concentration in the drop, the time between drop deposition and movement is reduced from ≈168 s for 0.5 M NaCF_3_SO_3_ to around 75 s for 1.25 M NaCF_3_SO_3_ in the absence of enforced airflow. With increasing airflow to 2000 and to 4500 rpm, the time between drop deposition and the initial drop movement decreased significantly. For an airflow of 2000 rpm (Figure 5a, red) and a concentration of 0.5 M, the drop starts moving after ≈70 s while this time further reduces to ≈25 s for 1.25 M NaCF_3_SO_3_. For an airflow of 4500 rpm (Figure 5a, blue), the drop starts moving already after about 37 s for a 0.5 M NaCF_3_SO_3_ solution drop and after about 16 s for a concentration of 1.25 M. Drop movement was as well observed for a 0.5 M aqueous solution of NaCF_3_COO and Mg(CF_3_SO_3_)_2_ (Figure S10), while no‐drop movement was observed for a solution of the surfactant sodium dodecyl sulfate (SDS, Figure S11a,b) or for an aqueous solution of NaCF_3_SO_3_ deposited on an oil‐filled mesoporous silica film (Figure S11c). Thereby, the drop of 0.5 M aqueous Mg(CF_3_SO_3_)_2_ solution started moving already after 7 seconds which is much faster than the drop of aqueous NaCF_3_COO or NaCF_3_SO_3_ solution, clearly indicating the influence of ion type on the drop movement. By increasing the airflow intensity from zero rpm to 4500 rpm, a decrease in time by factor ≈4 at 0.5 M NaCF_3_SO_3_ concentration and a decrease in time by factor ≈5 at 1.25 M NaCF_3_SO_3_ concentration were observed (Figure 5a black, blue). Thus, by increasing airflow to 4500 rpm and concentration from 0.5 to 1.25 M a decrease from 168 to 16 s was observed corresponding to a factor 10 earlier start of drop movement. This concentration and airflow‐dependent time to start drop movement further supports our hypothesis of evaporation‐induced flow and resulting asymmetric ion distribution within the drop driving drop movement.

In line with these observations, the speed of drop movement and thus the distance traveled by the drop increases with increasing NaCF_3_SO_3_ concentration in the drop (Figure 5b). At a constant airflow of 2000 rpm, a 1 µL 0.05 M NaCF_3_SO_3_ drop for example moves a distance of about 5 mm in 110 s (Figure S5) on a plasma‐activated mesoporous silica film. While increasing the concentration 15 times to 0.75 M, the drop moves a distance of 8 mm in about 67 s. An increase in ion concentration by a factor of 15 results in an increase in drop speed by a factor of 3. Further increasing the NaCF_3_SO_3_ concentration to 1.25 M increases the traveled distance to 20 mm in 47 s while as well the error bar representing the average values out of three measurements, increases. These observations imply that symmetry breaking probably by asymmetric evaporation and an asymmetric salt distribution is reached faster with increased concentration.

To correlate the drop speed with the related force, we measure the drop motive force (Figure 5c). The direction of the airflow was perpendicular to the drop and in plane with the sample surface. The drop adheres to the capillary force sensor and any additional motive force acting on the drop deflects it. Thus, the deflection of the force sensor balances the motive force. Without balancing the motive force, the drop would have moved otherwise in the presence of an airflow. We used 5 µL distilled (DI) water drops and 0.025, 0.1, 0.5, and 1 M aqueous NaCF_3_SO_3_ salt solution drops on plasma‐activated mesoporous films. The friction force along the sliding length remains constant for both, salts and distilled water (Figures S12 and S13). We measured increasing drop motive force with an increasing ion concentration of aqueous NaCF_3_SO_3_ solution (Figure 5d). In particular, we note an increase in motive force up to 0.5 M concentration. The motive force increases from ≈3 to ≈7 µN when the salt concentration is increased from 0.025 to 0.5 M. Thus, the force increased by a factor of 2. Increasing the concentration of aqueous NaCF_3_SO_3_ solution to 1 M does not increase the motive force further. The motive force (Figure 5d) is of a similar magnitude to the friction force of backward motion (Figure S6), indicating a movement on fluid filled mesopores. The error bars represent the variation in force across three independent drop motive force measurements on a sample. The scatter in data is high due to the vibration of the capillary in the presence of an airflow. Therefore, we conclude that higher the salt concentration of NaCF_3_SO_3_, faster and stronger the drop responds to an airflow which is in accordance with the speed and traveled distance of the drops.

To further understand the force for drop movement, we perform drop friction force measurements on mesoporous films (Figure 5e,f). A 5 µL drop of aqueous NaCF_3_SO_3_ or KCl salt solution was placed onto the mesoporous film. This drop is immobilized on the surface by a glass capillary equipped with a ring. The sample is then moved by the XY stage at two different velocities of 1 and 10 mm/s. We describe the forward motion of the drop when the drop moves over the surface for the first time. The forward motion revealed an average friction force in the range of 19 (±4) μN for the mesoporous film with a static CA of ≈10° at a moving speed of 1 mm/s (Figure 5f). This friction force is in accordance with our previous work on friction force in dependence of CA in which we detected a decreasing friction force with decreasing macroscopic advancing CA [73]. The measured force for a CA of ≈10° here is again lower. This is in accordance with the observation that this low CA is needed to observe drop movement (Figure 3). The friction forces for the speed of 1 mm/s are almost similar for both salts. For NaCF_3_SO_3_ drops, the friction forces are between 17 (±2) µN, while for KCl drops friction forces between 17 (±3) µN can be observed. With increasing speed of up to 10 mm/s, the friction forces increase to 35 (±3) µN for both salts (Figure 5b). Neither the salt type nor the salt concentration in the drop significantly affected the friction force, while salt type and concentration strongly affect programmable drop movement. This indicates that additional factors coupled to asymmetric evaporation are driving the movement itself.

## 4 Conclusion

We demonstrate programmable drop movement on hydrophilic mesoporous silica films upon symmetry breaking by a local airflow while using aqueous NaCF_3_SO_3_ salt solution. The nature of the ion and the surface determine if drop movement occurs. Based on the salt distribution and the airflow intensity‐dependent drop behavior, we hypothesize that the local airflow locally affects evaporation and induces flow within the drop resulting on one hand in a locally smaller imbibition ring at the side of the airflow and on the other hand on locally increasing salt concentration, including salt precipitation at the opposite side of the airflow visible after complete drop evaporation. This is in accordance with the literature investigating PEG containing water drops on polymer brush surfaces proposing a push‐pull mechanism, showing that the drop moves in the opposite direction to the flow [28]. In addition, in our experiments the applied salt is fluorinated which probably leads to local changes in surface properties upon salt enrichment and precipitation. All together this induces drop motion to the side opposite of salt precipitation and toward the airflow. To systematically investigate this hypothesis, local evaporation profiles, local time‐dependent salt distribution, and the flow dynamics within the drop in dependence of asymmetric airflow and evaporation will have to be recorded in future experiments. Nevertheless, the moving direction was precisely controlled through the airflow direction while the drop speed was controlled by the airflow intensity and the initial salt concentration within the drop. Drops can even be arrested suppressing drop movement by the right combination of substrate tilt angle and airflow intensity. Under these conditions, gravity is equilibrated by the force moving the drop toward the airflow. It has to be noted that the drops loose fluid due to evaporation and imbibition into the mesoporous layer during our experiments which may limit the application of the presented experimental conditions without further optimization. Nevertheless, a new mechanism of programmable drop movement using the inherent properties of a hydrophilic homogeneous mesoporous film was demonstrated. We expect these insights of programmable drop movement with salt solution on a hydrophilic mesoporous film to be of relevance for applications like water management, efficiency enhancement in water harvesting, surface patterning, or microfluidics.

## Funding

This work was supported by the Deutsche Forschungsgemeinschaft (AN1301/5, AN1301/8, BE 3286/6‐1, CRC 1194 C07, and CRC 1194 C04).

## Supporting information

Supplementary Material

## Data Availability

The data that support the findings of this study are available from the corresponding author upon reasonable request.
