# Supplementary material for: Directing Salt‐Drop Movement on Mesoporous Silica Films
Source: Small Sci. 2026 Jul 16;6(7):e70340. doi: 10.1002/smsc.70340 (PMC13387280; doi:10.1002/smsc.70340)
Supplement: Supplementary file 1 — Supplementary Material [file SMSC-6-e70340-s001.pdf]

Supporting Information

Directing salt-drop movement on mesoporous silica films

Laura Czerwenka[1], Chirag Hinduja[2], Lisa Balonier[1], Rüdiger Berger[2], Annette Andrieu-Brunsen[1]

[1]Ernst Berl Institute for Technical and Macromolecular Chemistry, Macromolecular Chemistry – Smart Membranes, Technical University Darmstadt, Germany

[2]Max Planck Institute for Polymer Research, Ackermannweg 10, 55128 Mainz, Germany

To verify that drop movement occurs exclusively on mesoporous surfaces, an aqueous 0.25 M KCl drop (Figure S1 a), an aqueous 0.25 M $NaCF_3SO_3$ (Figure S1 b) drop and an aqueous 0.1M $NaCF_3SO_3$ (Figure S1 c) drop were deposited onto a dense, non-porous silica film. No drop movement of the three salt solutions was observed until complete drop evaporation. In addition, no drop movement for 1 M $NaCF_3SO_3$ solution was observed on the silica dense films when a strong airflow was used with 4500 rpm (Figure S1c).

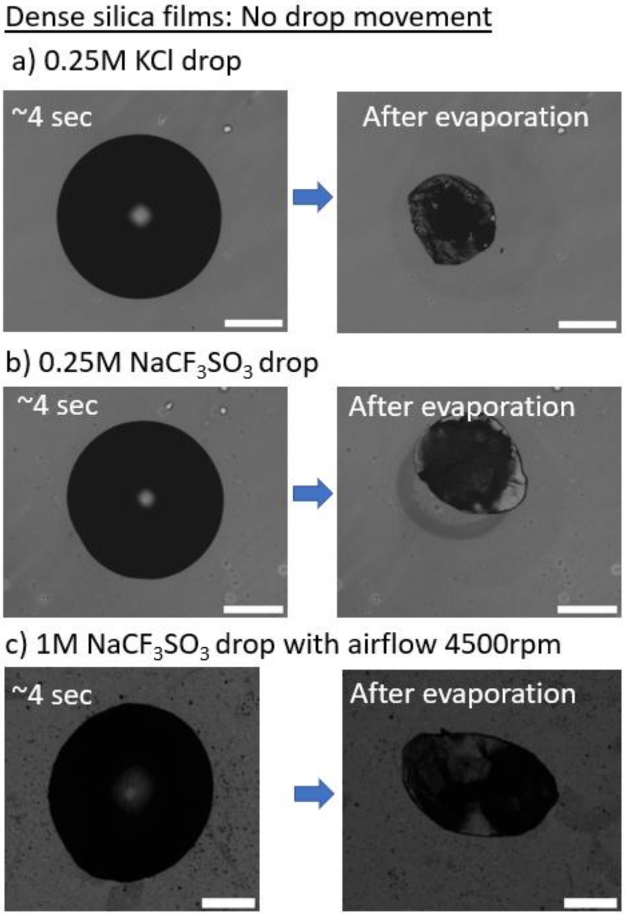

Figure S 1: a) 0.25M aqueous KCl drop, b) 0.25 M aqueous $NaCF_3SO_3$ drop and c) 1 M aqueous $NaCF_3SO_3$ drop with an airflow of 4500 rpm on dense coated silica film, showing no drop movement. Measurements were performed under constant humidity (~60%) and temperature (~23 °C).

Mesoporous films were incubated in 0.5 M $NaCF_3SO_3$ solution. Subsequently a drop of the same concentration was deposited onto the mesoporous film. The static CA of the incubated films was about 30°. The drop behavior was analyzed at two different air flow rates until complete drop evaporation. We observed no drop movement at air flow rates of either 2000 rpm or 4500 rpm. Instead, an asymmetrical salt distribution and an asymmetric imbibition ring were observed, similarly to the measurement with a KCl drop (Figure 3). This supports the hypothesis that the wettability of the film, the asymmetric evaporation and -salt distribution cause drop movement.

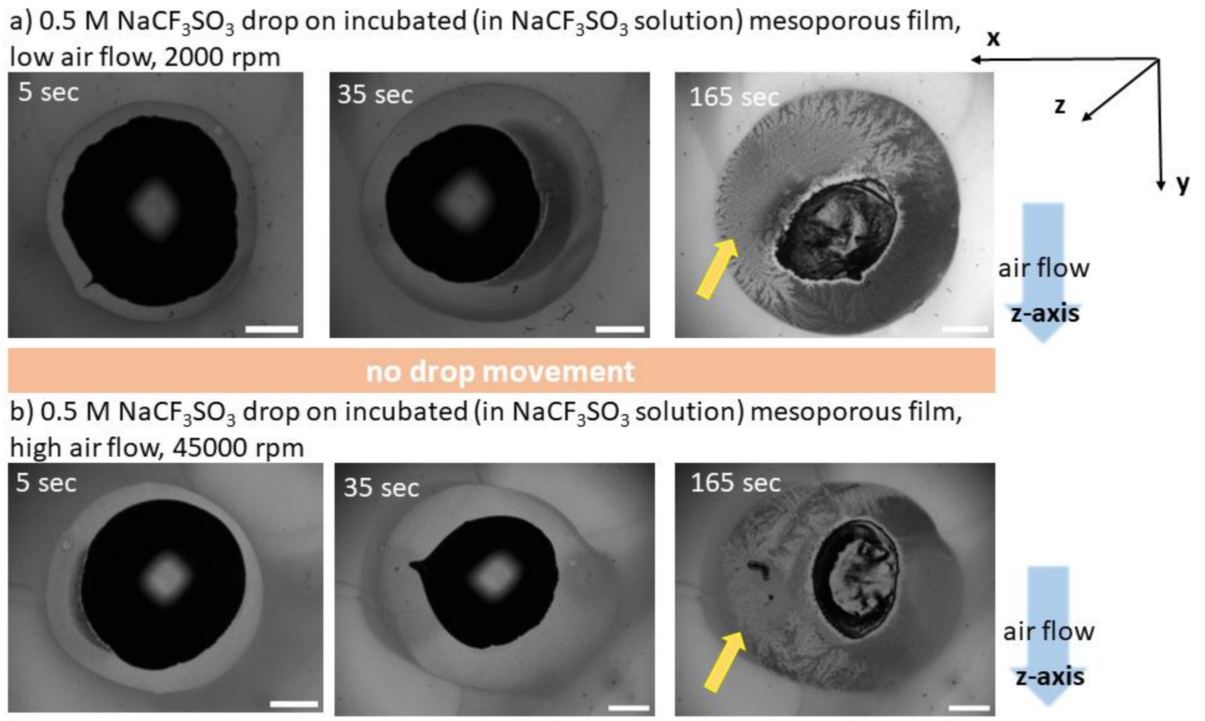

Figure S 2: No-drop movement was measured (expected in the direction of the x-axis) for 0.5 M $NaCF_3SO_3$ drop on incubated mesoporous silica film in $NaCF_3SO_3$ solution with a) low air flow (2000 rpm, z-axis direction) and b) high airflow (4500 rpm, z-axis direction) after 5, 35 and 165 seconds. Measurements were performed under constant humidity (~60%) and temperature (~23 °C).

Similar to the salt distribution of KCl and $NaCF_3SO_3$ (Figure S3), we present further images demonstrating the asymmetric salt patterns of the aqueous $NaCF_3SO_3$ solution after complete drop evaporation. The drops shown here exhibit increased salt precipitation on the side opposite the air flow, near the three-phase contact line or within the drop itself.

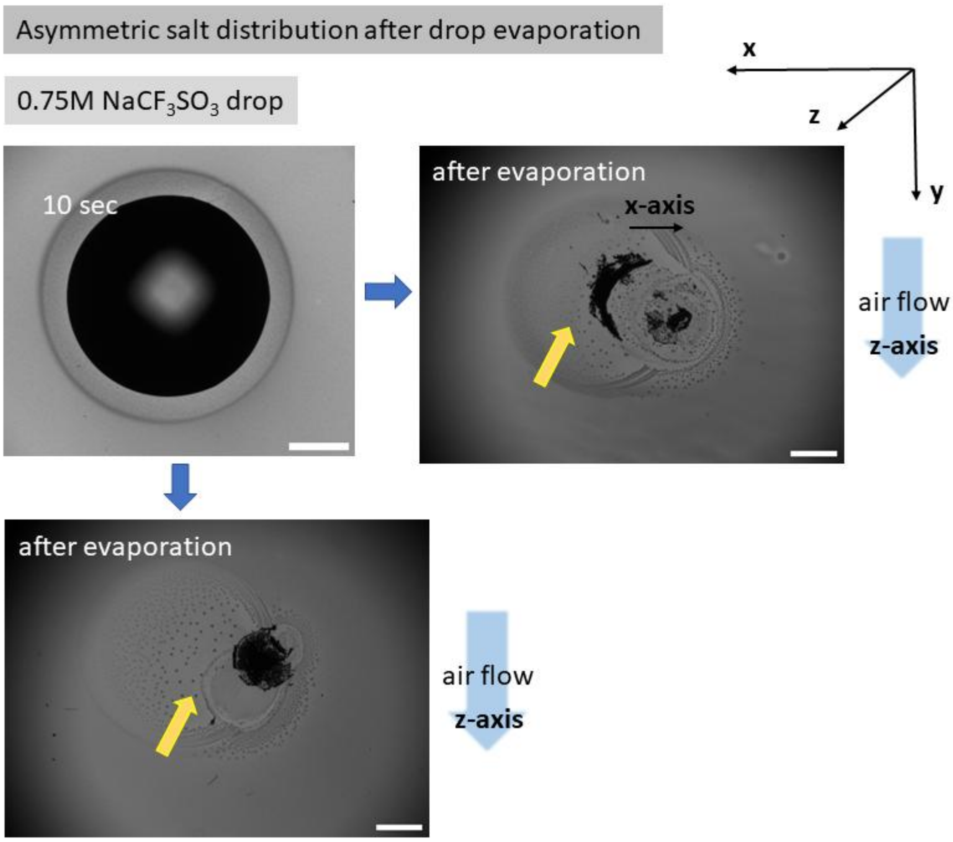

Figure S 3: Asymmetric salt distribution after drop movement in x-axis direction and complete drop evaporation for two different films measured with 0.75 M aqueous $NaCF_3SO_3$ solution on plasma activated mesoporous films with 2000rpm air flow intensity in z-axis direction. Measurements were performed under constant humidity (~60%) and temperature (~23 °C).

Tilting the mesoporous film at a certain angle changes the way the drop is transported due to gravity. This can be influenced by simultaneously using an asymmetric air flow through a fan (see Figure 4). When a tilt angle of 6° is combined with an airflow of either 2000 rpm or 4500 rpm, or when a tilt angle of 12° is combined with an airflow of 4500 rpm, the drop movement parallel to airflow away from fan position can be observed. Thus, gravity and the effect of the airflow balance each other.

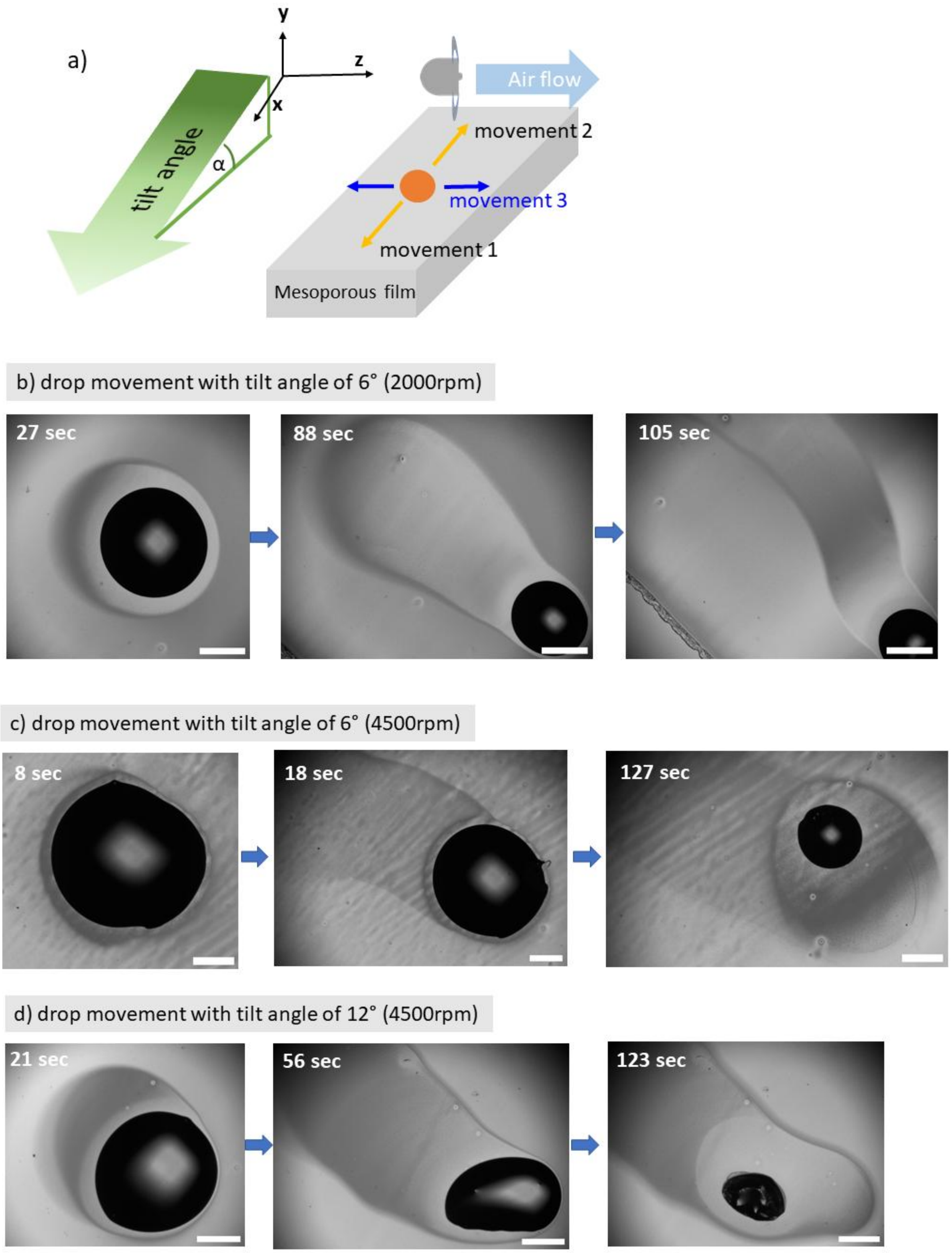

Figure S 4: a) Schematic representation of drop movement with different tilt angles α of the film which can change the drop movement direction from 1 or 2 to movement direction 3 when using a tilt angle of 6° in combination with 2000rpm and 4500rpm of airflow or when using a tilt angle of 12° of tilt angle in combination with 4500rpm of airflow. The airflow direction is defined as the z-axis. This drop movement direction parallel to the airflow (movement 3 in the scheme) was only observed in this special situation around the transition tilt angle for some droplets. For lower tilt angles the drops consistently move uphill while for higher tilt angles the droplets move downhill. b) 0.5 M $NaCF_3SO_3$ drop measured with tilt angle of 6° and an air flow of 2000 rpm, c) with a tilt angle of 6° with an airflow of 4500 rpm and d) with a tilt angle of 12° with an airflow of 4500 rpm on mesoporous films. Measurements were performed under constant humidity (~60%) and temperature (~23 °C). Scale bar is 1mm.

With increasing $NaCF_3SO_3$ drop concentration, the measurement time required for the drop to move a certain distance decreases. Thus, with increasing ion concentration the drop moves a longer distance (Figure 5b) in a shorter time (Figure S5).

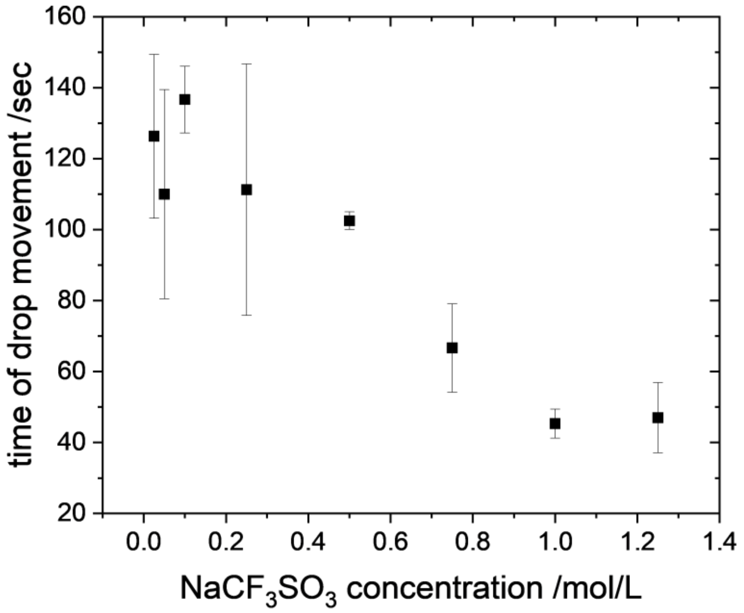

Figure S 5: Time required for the drop to travel a specific distance, depending on the $NaCF_3SO_3$ concentration in the drop. This distance used to determine the speed varied between different drops. The data is related to the speed of drop movement shown in Figure 5b.

Based on previous work[1], the speed of drop movement of 10 mm/s in forward motion (Figure 5f) is expected to be faster than the speed of capillary imbibition of the fluid into the mesoporous film forming the imbibition ring around the drop. Consequently, with a drop speed of 10 mm/s in the forward direction, the drop is expected to slide on an air-filled mesoporous layer and not on a fluid-filled mesoporous layer which is consistent with the observed higher friction force. Interestingly, the friction force is predominantly depending on the mesoporous film characteristics, such as its wettability, and the speed of drop movement.

When the drop traverses back along the same path, we describe it as a backward motion (Figure S 6). The backward drop movement occurs on liquid filled pores as the liquid remains in the pores after the forward movement. An almost similar friction force for both speeds (1 mm/s and 10 mm/s) can be observed for backward motion, compared to forward motion. A difference in the friction force between the two speeds with maximum 5 (±2) µN for $NaCF_3SO_3$ and 3 (±8) µN for KCl solution for the backward motion and a maximum force of 23 (±2) µN for $NaCF_3SO_3$ and 20 (±8) µN for KCl solution for the forward motion were measured. We assume that the differences in speed (1 mm/s and 10 mm/s) are smaller for the backward motion because the drop movement is based on fluid-filled pores and therefore the imbibition time and also the drop volume required for imbibition are reduced. For backward motion we observed a lower friction force for a $NaCF_3SO_3$ solution (Figure S 6, orange) compared to KCl solution (Figure S 6, pink). For 500 mM KCl solution a higher friction force by a factor of 8 can be observed, while the friction force for 500 mM $NaCF_3SO_3$ solution is almost 0.

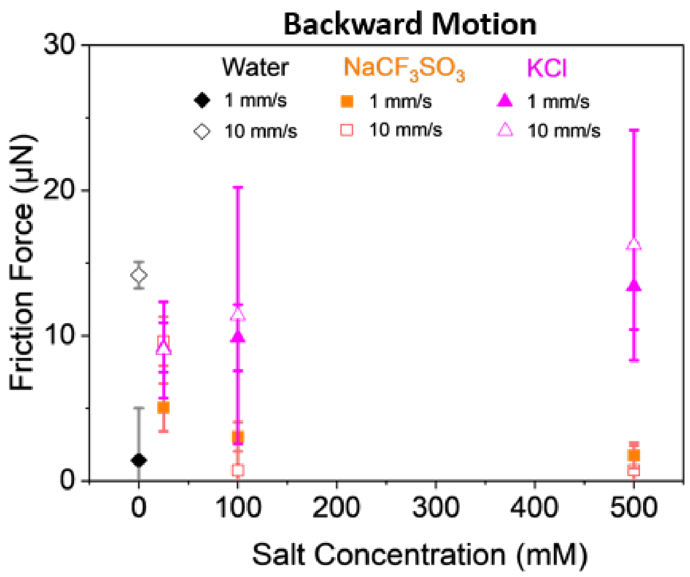

Figure S 6: Drop friction force measurement of a 5 µL water or aqueous salt solution ($NaCF_3SO_3$ or KCl) drop is placed on the capillary and moved back on top of the mesoporous film. Average kinetic friction force during backward motion of a 0.025 M, 0.1 M and 0.5 M KCl (pink)-, $NaCF_3SO_3$ (orange) and water (black) drop on mesoporous silica film. The standard deviation was represented by the error bars and was determined from two individual drops measured on one mesoporous substrate. The error bars represent the statistical variations of force in the kinetic region within a scanline.

Figure S7 shows the experimental setup used to record droplet motion at various airflow intensities and $NaCF_3SO_3$ concentrations.

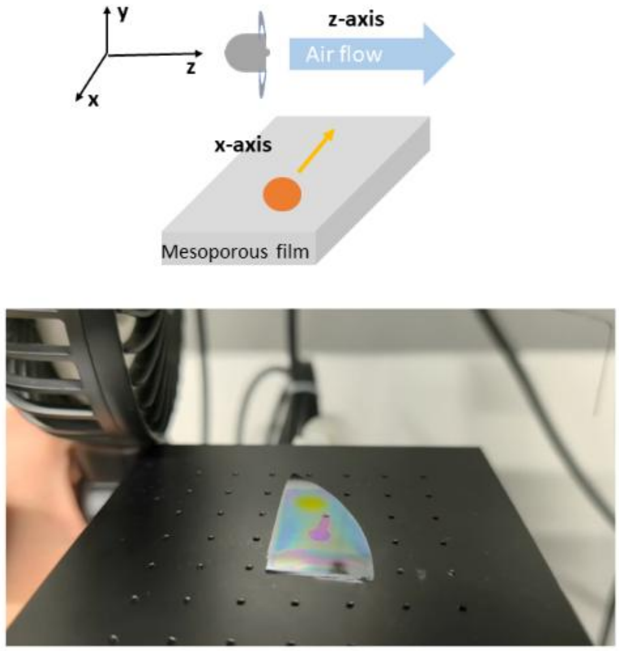

Figure S 7: Position of the fan (airflow) relative to the substrate. Scheme in comparison to picture of the setup with a 0.5 M aqueous $NaCF_3SO_3$ solution drop trace being optically visible. Experimental conditions: 4500 rpm and plasma activated mesoporous film.

For drop movement, CA -and DoFFi measurements we used the following solution composition:

Table S 1: Aqueous solutions for different concentrations for drop movement, CA -and DoFFi measurements.

$NaCF_3SO_3$						
M / g mol^{-1}	m / g	n / mol	V / L	pH	drop of acid	c [mol/L]
172.1	0.430	0.0025	0.025	8	0	0.1
172.1	0.108	0.0006	0.025	8	0	0.025
172.1	0.215	0.0013	0.025	10	0	0.05
172.1	1.075	0.0063	0.025	11	0	0.25
172.1	2.151	0.0125	0.025	11	0	0.5
172.1	3.226	0.0188	0.025	12	0	0.75
172.1	4.302	0.025	0.025	12	0	1
172.1	5.377	0.0313	0.025	12	0	1.25
KCl						
M / g mol^{-1}	m / g	n / mol	V / L	pH	drop of acid	c [mol/L]
74.6	0.186	0.0025	0.025	7	0	0.1
74.6	0.093	0.0013	0.025	7	0	0.05
74.6	0.932	0.0125	0.025	7	0	0.5
$KClO_4$						
M / g mol^{-1}	m / g	n / mol	V / L	pH	drop of acid	c [mol/L]
138.6	0.346	0.0025	0.025	7	0	0.1
138.6	0.173	0.0013	0.025	7	0	0.05
138.6	1.732	0.0125	0.025	7	0	0.5
K_3PO_4						
M / g mol^{-1}	m / g	n / mol	V / L	pH	drop of H_3PO_4	c [mol/L]
212.3	0.531	0.0025	0.025	6	2	0.1
212.3	0.265	0.0013	0.025	7~8	4	0.05
212.3	2.653	0.0125	0.025	8	14	0.5
$Mg(CF_3SO_3)_2$						
M / g mol^{-1}	m / g	n / mol	V / L	pH	drop of acid	c [mol/L]
322.4	0.806	0.0025	0.005	10	0	0.5
$NaCF_3COO$						
M / g mol^{-1}	m / g	n / mol	V / L	pH	drop of acid	c [mol/L]
136.0	0.340	0.0025	0.005	7	0	0.5
SDS						
M / g mol^{-1}	m / g	n / mol	V / L	pH	drop of acid	c [mol/L]
288.4	0.144	0.0005	0.005	-	0	0.1
288.4	0.721	0.0025	0.005	-	0	0.5

We measured the airflow using a thermal anemometer 405i (TA) 0-30 m/s measuring range (Wirth Klimatechnik) with fixed distances in climate room with 52.1 RH%, 23 °C.

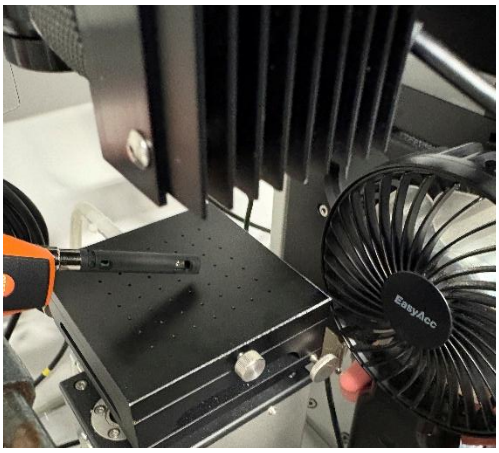

Figure S 8: Measurement of velocity with ~4cm distance and thus at the position of the sample with respect to the fan.

Table S 2: Air flow velocity with ~ 4cm distance at the sample position with airflow 2000rpm and 4500rpm. Same setup as in Figure 2-5b.

airflow in rpm	velocity in m/s
2000	0.21
4500	0.30

Direct measurement of velocity using a varying distance of fan:

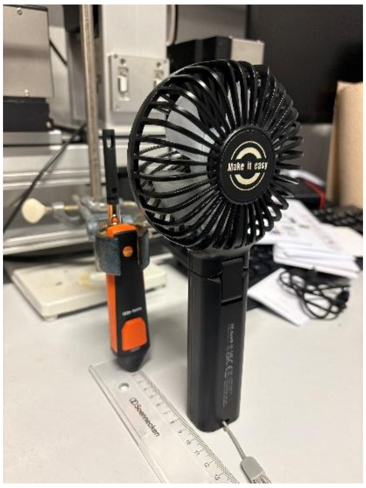

Figure S 9: Experimental setup for direct velocity measurements with varying distance.

Table S 3: Measurement of air flow velocities with two different airflows at 2000rpm and 4500rpm along the airflow axis (z-axis) at different distances from the fan.

Airflow [rpm]	2000	2000	2000	2000	2000	2000
distance [cm]	3	6	9	12	15	18
velocity [m/s]	2.91	2.04	1.51	1.10	1.48	0.86
Airflow [rpm]	4500	4500	4500	4500	4500	4500
distance [cm]	3	6	9	12	15	18
velocity [m/s]	5.65	5.29	3.80	3.93	3.23	2.35

Furthermore, additional experiments using 0.5M $NaCF_3COO$ and $Mg(CF_3SO_3)_2$ salt solutions (Figure S10 a) and b)) as well as sodium dodecyl sulfate (SDS, Figure 11) as surfactant were carried out. Using $Mg(CF_3SO_3)_2$ we observe that the drop starts moving much faster similar to an increasing concentration when using $NaCF_3SO_3$ (Figure 5 a) blue). Using SDS no drop movement was observed. Consequently, we hypothesize that the salt nature together with local evaporation induced flows and local salt precipitation cause the observed drop movement.

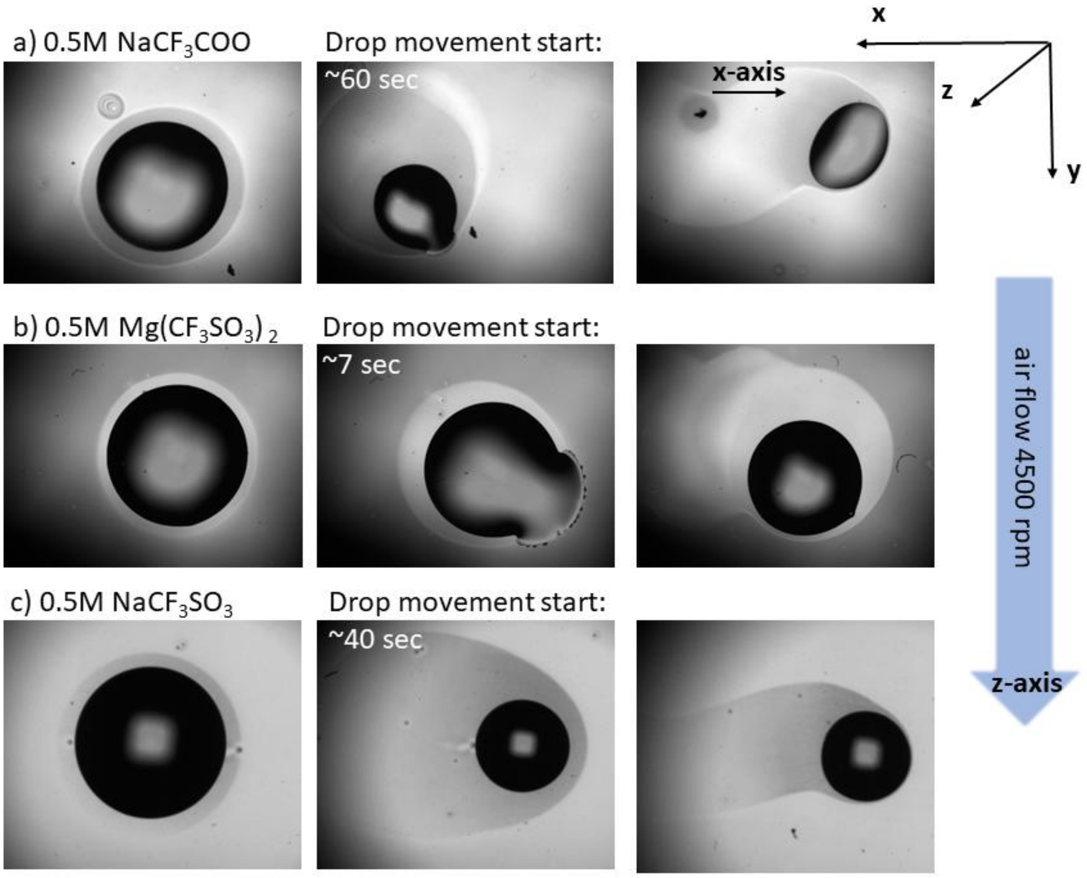

Figure S 10: Start time of drop movement on plasma activated mesoporous silica films with 1µL 0.5M aqueous salt solution drop using a) $NaCF_3COO$, b) $Mg(CF_3SO_3)_2$, c) $NaCF_3SO_3$ and 4500 rpm airflow intensity. The air flow was directed along the z-axis while the drop moved orthogonally towards the airflow along the x-axis. The time needed between drop deposition and drop movement is salt type dependent and concentration dependent. Thereby, $Mg(CF_3SO_3)_2$ shows the fastest start of drop movement already after ~ 7 seconds, being even faster than the highest $NaCF_3SO_3$ concentration of 1.25 M under similar conditions needing 17 seconds to start drop movement (Figure 5a).

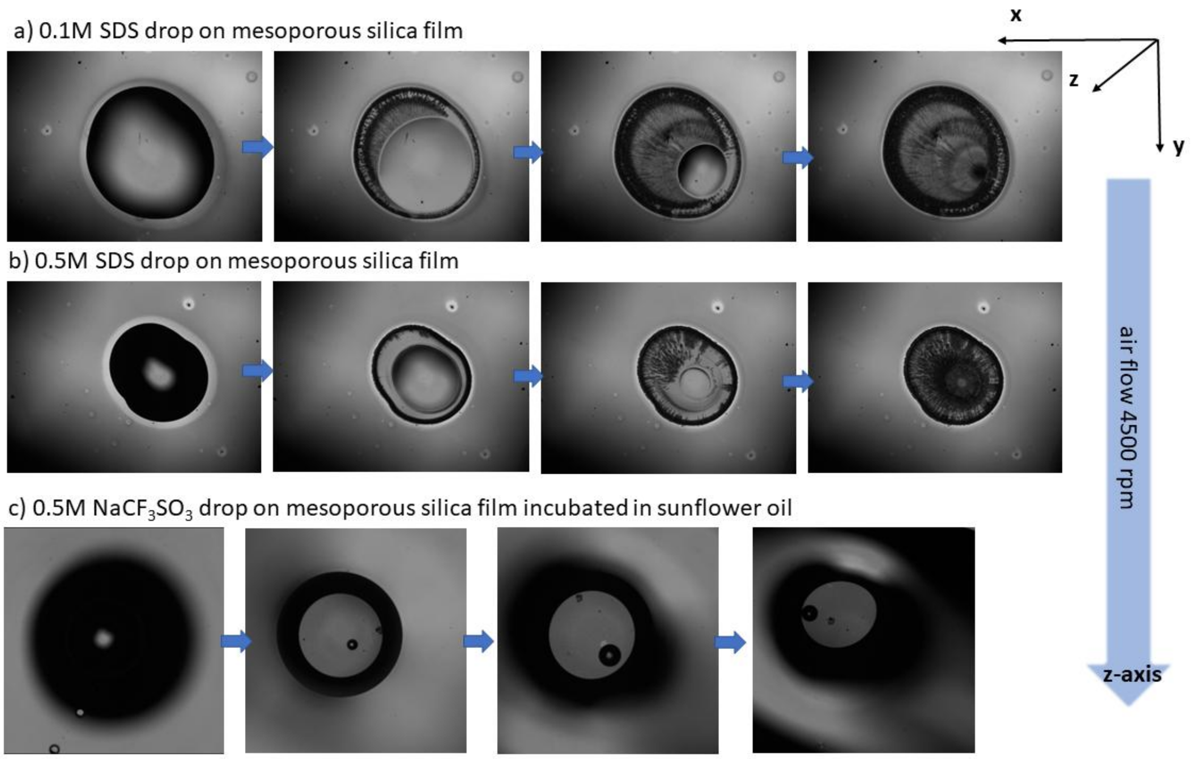

Figure S 11: No drop movement was observed for a) 0.1M SDS drop, b) 0.5M SDS drop on mesoporous silica films and c) 0.5M NacF$_3$SO$_3$ drop on mesoporous film incubated in sunflower oil for 1 h using 4500 rpm airflow intensity in the direction of z-axis.

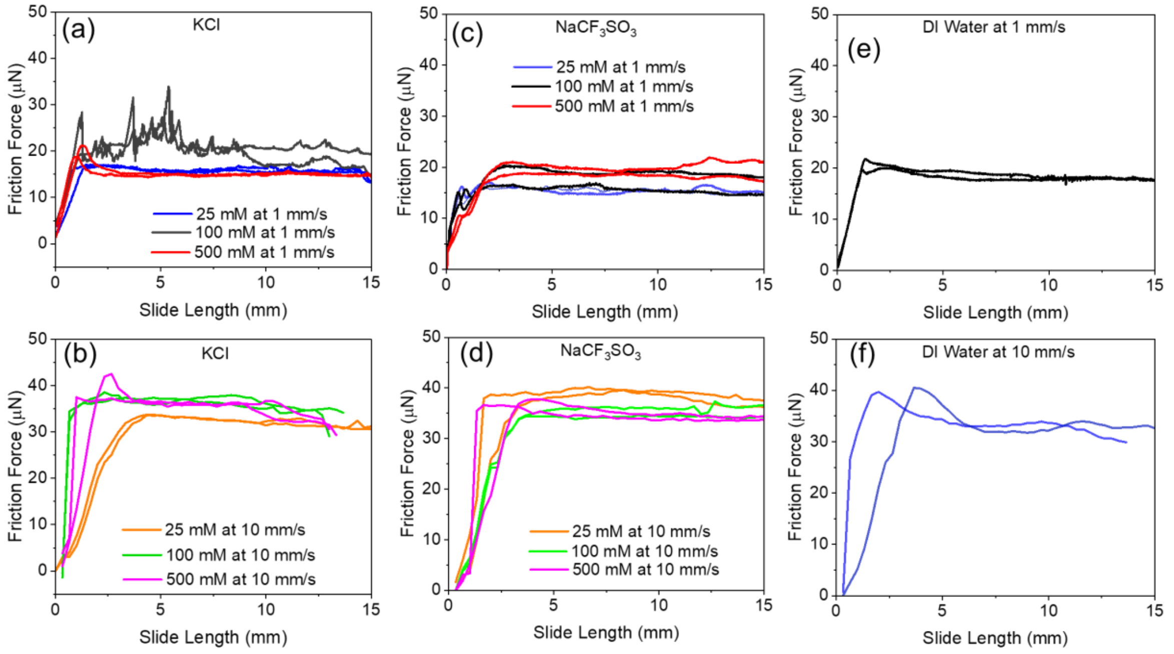

Figure S 12: Friction force measurements along the sliding length.

Regarding measurements of contact angle (CA) hysteresis from DoFFI data: the advancing CA of a sliding drop on these mesoporous films is ~ 34°. However, the receding CA is immeasurable due to formation of a thin liquid film at the rear. Please see below an instant (an image) from our $NaCF_3SO_3$ drop sliding DoFFI experiment at 1 mm/s speed. The thin liquid film at rear indicates almost zero receding CA. Thus, making CA hysteresis measurement impossible. This inability to measure receding CA was our primary motivation to measure drop friction force instead of CA hysteresis.

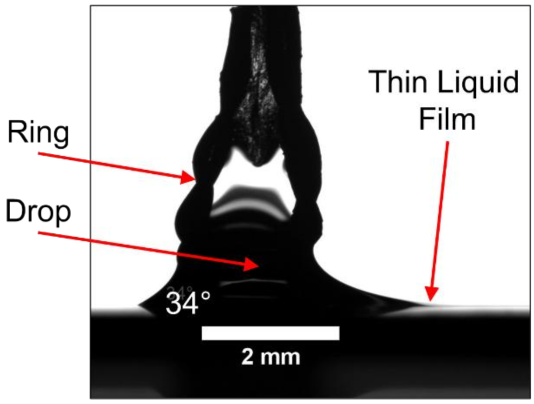

Figure S 13: The advancing CA of a sliding drop on these mesoporous silica film (without plasma treatment).

Measurements were taken of the drop movement of a 0.5 M aqueous solution of $NaCF_3SO_3$ towards an airflow of 4500 rpm on mesoporous silica films at tilt angles of 1–5°.

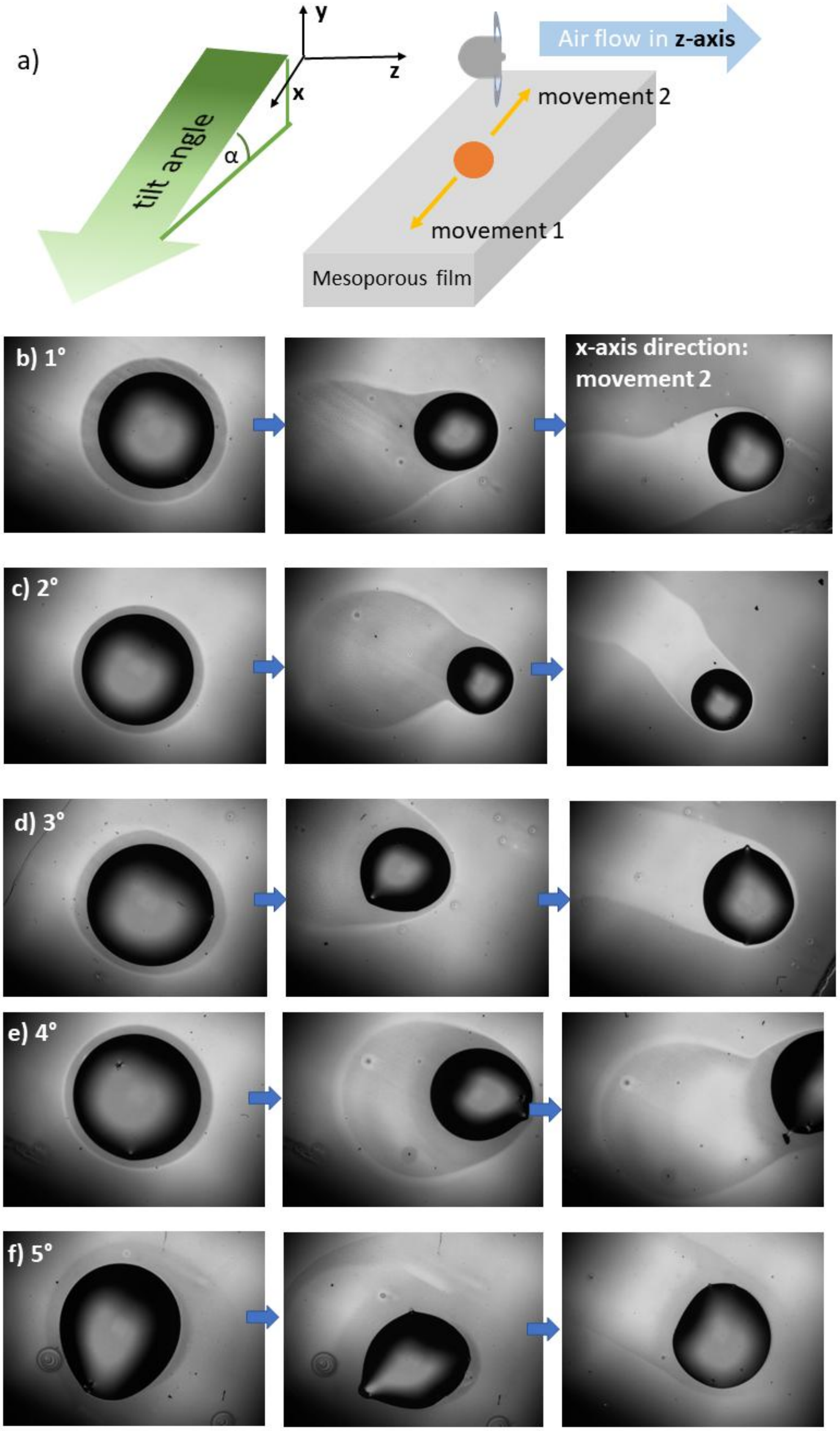

Figure S 14: a) Schematic representation of drop movement perpendicular to the airflow with different tilt angles α of the film with b) 1°, c) 2°,d) 3°, e) 4° and f) 5° and an airflow intensity of 4500 rpm on mesoporous films. When the airflow direction is defined as the z-axis, all the drops shown here move along the x-axis toward the airflow and uphill. This corresponds to the drop movement 2 in the scheme. Measurements were performed under constant humidity (~60%) and temperature (~23 °C).

As experimentally demonstrated in Figure S14 uphill motion is observed for tilt angles of 1-5°. Below, we made approximate calculations to predict a tilt angle where uphill drop motion is possible.

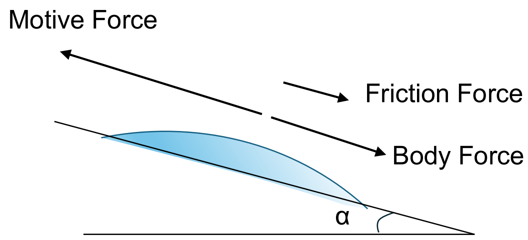

Figure S 15: Schematic representation of drop movement with tilt angle α.

The drop can move uphill when the motive force is higher than the combined resistance of body force and friction force. In our experiment, we observe the highest motive force for a 5 µL drop of $NaCF_3SO_3$ 500 mM salt at 4500rpm, which is 7 µN (Fig. 5d). We keep these parameters fixed and determine the tilt angle at which this drop can slide uphill. CAs of the drop correspond to a sessile drop as shown in Figure 3f of the main manuscript. Therefore, the advancing CA (θ_a) can be taken as ~8-10° for plasma treated mesoporous films and the receding CA (θ_r) = 0°. The receding CA is taken zero since the drop forms a thin liquid film at its rear (Figure S13). With the known advancing and receding CA, we estimate the friction force (F) from Kawasaki-Furmidge equation, which is the following expression:

$$F = k\gamma w(cos\theta_r - \ cos\theta_a)$$

Here, $k \leq 1$ is a numerical constant. We assume $k = 1$ for simplification. $k = 1$ will yield higher value of friction. γ is the surface tension of the liquid drop, which we assume close to water = 72 mN/m for $NaCF_3SO_3$ 500 mM drop. w is the width of the drop. Since the contact angles of sessile drops are low on mesoporous surfaces, indicating that the drop spreads on such surfaces. Therefore, from the above sliding drop image (Figure S13) we assume length ≈ width (w) ≈ 3 mm.

Now applying the condition for uphill motion:

Motive force > Body force + friction force

$$7\ \mu N > (mgsin\alpha) + \gamma w(cos\theta_r - \ cos\theta_a)$$

$$7\ \mu N > (5\ \mu L \times 1000\ Kg/m^3 \times 10 m/s^2 \times Sin\alpha) + (72\ mN/m \times 3\ mm \times (\cos 0° - \cos 8°)$$

Solving for α yields:

$$\alpha < 5.6°$$

Therefore, our quick estimation predicts that the uphill drop movement is possible if we take a 5 µL drop of 500 mM $NaCF_3SO_3$ salt and with fan running at 4500 rpm. We may see the uphill drop motion for surface tilt angle of less than 6°. This coincides well with our experiments showing uphill movement for tilt angles of 1-5 ° (Figure S14).

Literature

[1] Laura Czerwenka, Annette Andrieu-Brunsen Self-Driven Fluid Imbibition of Salt Solutions into Mesoporous Films, Langmuir **2025,** DOI 10.1021/acs.langmuir.5c00650.
